# Evaluation of the microbial tightness of closed system transfer devices by simulating airborne and touch contamination

**DOI:** 10.1186/cc14165

**Published:** 2015-03-16

**Authors:** J Gebel

**Affiliations:** 1 University of Bonn, Germany

## Introduction

The use of intravascular catheter devices is often associated with serious bloodstream infections due to microbial contaminations. To minimize risk of such infections NIOSH recommends the use of closed system transfer devices (CSTDs). To evaluate the microbial tightness of CSTDs we developed two methods which simulate the bioburden in ambient air of operating rooms and ICUs.

## Methods

The methods simulate airborne and touch contamination. We tested the microbial tightness of the integrated Safeflow^® ^valve of a Mini-Spike^® ^which is used for drug admixture. The airborne contamination was done in an exposure chamber in which a nebulizer distributed defined *B. subtilis *spore aerosols [1]. A Mini-Spike^® ^was inserted into a vial of 0.9% sodium chloride solution (NaCl). A nebulizer with a suspension of 4.8 × 10^5 ^CFU spores of *B. subtilis *per ml was used to generate an aerosol for 1 minute. The volume of *B. subtilis *suspension nebulized per minute was 0.278 ml. This corresponds to 1.34 × 10^3 ^aerosolized spores in the exposure chamber, which has a volume of 0.24 m^3 ^(5.6 × 10^3 ^CFU per m^3 ^air). The used concentration was 100 times higher than the microbial burden found in hospitals [2]. After nebulization the valve was disinfected and NaCl was withdrawn into a syringe at certain time intervals. The NaCl was incubated on tryptic soy agar at 37°C for 48 hours. Results were documented as CFU. For touch contamination, a Mini-Spike^® ^was attached to a vial of NaCl. The valve of the Mini-Spike^® ^was contaminated with 10^5 ^CFU *Staphylococcus aureus*. The subsequent procedure was done as described above.

## Results

Out of nine tested valves, none showed transmission of *B. subtilis *spores after airborne contamination. Three out of nine tested valves were contaminated with *S. aureus *after touch contamination.

## Conclusion

Our study shows that both Methods are suitable for evaluating the microbial tightness of CSTDs.
